# The development of variable system-based internet of things for the solar greenhouse and its application in lettuce

**DOI:** 10.3389/fpls.2024.1292719

**Published:** 2024-01-29

**Authors:** Lingzhi Li, Furong Han, Jingjing Li, Shunwei An, Kaili Shi, Shirui Zhang, Lili Zhangzhong

**Affiliations:** ^1^ College of Horticulture, Shanxi Agricultural University, Shanxi, China; ^2^ Beijing Research Center of Equipment Technology, Beijing Academy of Agriculture and Forestry Sciences, Beijing, China; ^3^ Agricultural Extension Station, Beijing Municipal Bureau of Agriculture and Rural Affairs, Beijing, China; ^4^ Key Laboratory for Quality Testing of Software and Hardware Products on Agricultural Information, Ministry of Agriculture and Rural Affairs, P.R., Beijing, China

**Keywords:** solar greenhouse, east-west ridge orientation, variable irrigation, internet of things (IoT), water management

## Abstract

The east-west ridge orientation has recently become an important agronomic method to improve mechanization in solar greenhouses. However, these ridge orientation changes shape differences between different ridges in crop water consumption, and there is a lack of research on the regulation and adaptation of water consumption. Therefore, this study introduces a variable irrigation decision-making method based on the Internet of Things management and control system for an east-west ridge orientation. To replenish water on demand, this study seting the variable irrigation decision-making (VRI) methods and traditional average irrigation decision-making (URI) methods in the system, and lettuce cultivation experiments were conducted to verify the effectiveness of the model and system. The results show that the difference of accumulated photosynthetically active radiation is the most significant between different ridges of the east-west ridge orientation, and the coefficient of variation is 43.77 %, which can be used as an activating factor for VRI methods. The irrigation water consumption, yield, water-use efficiencies, and irrigation water utilization of lettuce at different levels of irrigation were 307.12 L/m^2^, 5854.07 kg·ha^-1^, 1391.47 kg·ha^-1^·mm^-1^, and 7.63 kg·cm^-3^, respectively. Compared with the URI methods, the VRI method saved 10.02 % of water, increased yield by 9 %, and enhanced water use efficiency and irrigation water use efficiency by 12 % and 21.32 %, respectively. This study provides a new approach for improving crop production efficiency under an east-west ridge orientation.

## 1 Introduction

The United Nations Food and Agriculture Organization (FAO) survey results show that the world is worried about future food demand, and the world’s population is expected to reach 9.73 billion by 2025 (FAO, 2017). In addition, sudden weather changes and water shortages have increased pressure on food production. Facility agriculture has become an important source of agricultural products for urban and rural residents. Therefore, it is considered a solution to ensure food security and sustainability (Rayhana et al., 2020). China has the largest agricultural facility area in the world. The total area of horticultural facilities was more than 3.9 million hectares in China, accounting for more than 80% of the total area of horticultural facilities worldwide in 2022 (Sun et al., 2019). Solar greenhouses are the main type of facility and accounting for more than 70% of the total in China. Most solar greenhouses to date face south, and the planting mode is mainly along the north-south ridge orientation with long and numerous characteristics. The average solar greenhouse is usually 50–100 m long and 6–12 m wide. As a result, the length and number of ridges in north-south ridge orientation are 0.1–0.12 times and 8.75–8.88 times more than those in east-west ridge orientation, respectively.

Sustainable mechanized operation is challenging, Because mechanical work must be turned around frequently in the north-south ridge orientation, (Yang et al., 2021a; Yang et al., 2021b; Yang et al., 2022). As the population grows and ages, the number of people engaged decreases, increasing labor costs in agriculture. Therefore, it is important to improve the mechanized operations of solar greenhouses. China has started to develop an east-west ridge orientation instead of the north-south ridge orientation to lengthen the ridge, reduce the number of ridges, and improve the degree of mechanization in the solar greenhouses (Song, 2018). Studies have shown that the mechanization efficiency of ridge breaking is 14 times higher than that of manual labor, the soil crushing rate is up to 90%, and the cost savings are 13 times greater than those of manual labor (Wang et al., 2023). Another study compared the effects of east-west and north-south ridge orientations on tomato yield in a sliding solar greenhouse and reported that the yield of tomatoes in the east-west ridge orientation was higher than that in the north-south ridge orientation (Yang et al., 2020). Similarly, Na Liu et al. showed that the yield of machine-planted chili peppers was 2380 kg, which was 11.21% higher than that in the east-west ridge orientation planting (Liu et al., 2021). Yang Yandong et al. planted tomatoes with the same density and different ridge distances in the east-west ridge orientation. The results showed that increasing the ridge orientation was beneficial for increasing light transmittance, and yield and fruit quality were improved (Yang et al., 2021a; Yang et al., 2021b; Yang et al., 2022). The planting density differed in the east-west ridge orientation, and the light transmittance range of the tomato population increased with decreasing density (Yang et al., 2021a; Yang et al., 2021b; Yang et al., 2022). In summary, the variable irrigation decision-making method has been applied in many areas, such as Ningxia and Shenyang, and has become a main development direction.

Solar greenhouses shield against rainfall; therefore, irrigation is the only water source for crops. Water regulation is key for promoting crop growth and utilization. There are differences in water use characteristics of crops (Zhang et al., 2023). However, existing research has mainly focused on the downward planting density of the east-west ridge orientation and the changes in the crop growth environment caused by the change in ridge orientation; however, there is a gap in research on precise water control. In actual production, the average irrigation of the entire greenhouse limited the increase in production capacity, which follows the traditional north-south ridge orientation. Many studies have shown that variable irrigation can improve crop yield, quality, and water-use efficiency, while reducing agricultural water consumption. Previous studies have reported that intelligent irrigation management strategies can reduce irrigation water consumption by 13% without affecting yield or quality (Touil et al., 2022). Other studies have demonstrated that variable irrigation increases crop yield and improves water use efficiency better than uniform irrigation (Sui and Yan, 2017). Liakos et al. identified that the UGA smart sensor array is highly compatible with the VRI system, which kept the soil moist without affecting crop growth and used 25% less irrigation water than in traditional methods (Liakos et al., 2017). In summary, variable-rate irrigation is expected to become an important way to improve productivity under east-west ridge orientation in solar greenhouses.

In this study, a VRI method and an Internet of Things (IoT) management and control system are proposed for east-west ridge-oriented planting in solar greenhouses. The purpose of this study was to (1) determine whether the distribution of environmental parameters in solar greenhouses affects crop growth and water consumption in east-west ridge-oriented planting to provide a basis for regional irrigation and identify top influencing factors; (2) develop a VRI method driven by accumulated photosynthetic active radiation and an IoT management of control system to carry out zonal management according to the law of light distribution, arrange the variable irrigation system, and control the internal operation of the system by an algorithm; and (3) compare the effects of VRI methods with those of traditional URI methods on water consumption, growth rate, photosynthetic parameters, yield, water use efficiency, and irrigation water use efficiency in Loose leaf lettuce planted in the east-west ridge orientation.

## 2 Materials and methods

### 2.1 Intelligent variable irrigation system setup

**Figure 1:**
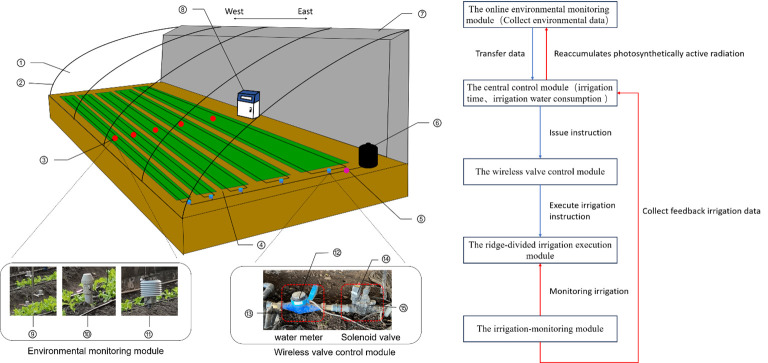
On the left is the Intelligent irrigation system detailed diagram. On the right is the Intelligent irrigation system workflow The blue and red lines represent the execution mechanism and the feedback mechanism, respectively. Circled numbers indicate unique parts of the system: ① solar greenhouse, ② shelter membrane, ③ instrument placement, ④ East-West ridge planting, ⑤ pressure gauge, ⑥ bucket, ⑦ rear wall, ⑧ central control cabinet, ⑨ photosynthetic radiation sensor, ⑩ soil moisture sensor, ⑪ greenhouse environment sensor, ⑫ water meter, ⑬ wireless collector ⑭, wireless valve controller, and ⑮ solenoid valve.

**Figure 1** shows the proposed intelligent variable irrigation system for a solar greenhouse and its workflow. The system comprises an environmental online monitoring module, a central control module, a wireless valve control module, a ridge irrigation execution module, and an irrigation monitoring module. The functions of each module are as follows:

(1) The online environmental monitoring module includes greenhouse multiparameter sensors that monitor air temperature, air humidity, and photosynthetic active radiation in the solar greenhouse. The collection frequency of air temperature and humidity was 1 h/times, and the collection frequency of photosynthetically active radiation was 5 min/times. The collected data were transmitted to the central control module through a wired network. The greenhouse multiparameter sensor was set 20 cm above the crop canopy, and its height was adjusted with the growth of the crop.
(2) The central control module first receives the data transmitted by the environmental online sensor, determines the irrigation time by accumulating photosynthetically active radiation, irrigation water consumption was calculated *the Penman-Monteith (modified) formula* (Allen et al., 1998), and issues the instructions of irrigation (*QNopen* and *QNstop*) to the wireless valve control module via wireless network transmission.
(3) The wireless valve control module consists of a wireless valve controller and a solenoid valve, which receives relevant commands from the central control module via wireless network transmission and executes the irrigation instructions(*QNopen* and *QNstop*) of the ridge irrigation execution module through the solenoid valve.
(4)The ridge-divided irrigation execution module includes the main pipe, sub-main pipe, and drip irrigation belt at the end, which are arranged in an east-west ridge orientation; the water is transported to the crop root through the pipeline and drip irrigation belt. The ridging layout is shown in **Figure 1**. The environmental online monitoring module is set up on each ridge in the green area, which is controlled by the central monitoring module. The entire study area is divided into ridges according to environmental parameters.
(5) The irrigation-monitoring module includes a wireless collector, water meter, and pressure gauge. A water meter and wireless collector were installed to measure the irrigation water consumption of each ridge at the back end of the solenoid valve, and a pressure gauge was installed on the main road.

**Figure 2:**
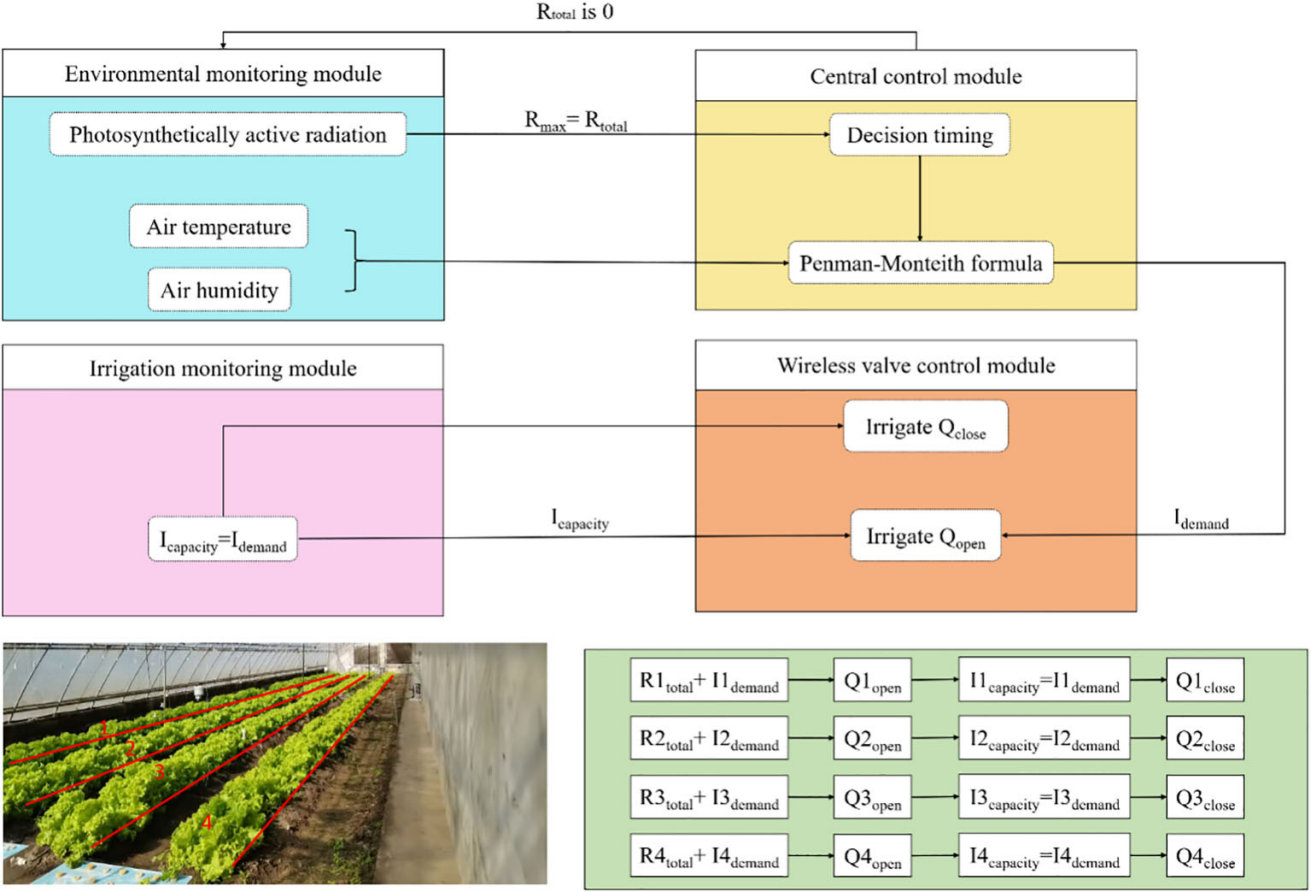
Irrigation decision model logical flow diagram with ridge splitting.

An intelligent variable-irrigation system was built around the VRI model. The underlying logic of the VRI model is detailed in **Figure 2**, in which the light heterogeneity that exists under east-west ridge orientation planting is used to delineate the area. Meanwhile, real-time collection of photosynthetic active radiation was performed to determine the irrigation time and consumption of water using *the Penman-Monteith (modified) formula* to achieve dynamic and accurate replenishment of water. The resulting instruction abbreviations are shown in **Table 1**.

**Table 1:** Abbreviated instruction summary table.

*QNopen*: Open irrigation	*RNmax*: The system sets the cumulative photosynthetically active radiation value	*INdemand*: The amount of water that crops theoretically need for irrigation
*QNstop*: Stop irrigation	*RNtotal*: The cumulative photosynthetically active radiation value of the system through environmental monitoring	*INcapacity*: Irrigation water use monitored by the system

First, the photosynthetically active radiation sensor receives light in the solar greenhouse and measures its cumulative photosynthetically active radiation (*RNtotal*) based on the density of the light quantum flux. *RNtotal* is then used to dynamically collect and calculate the hourly rate for each ridge, VRI1, VRI2, VRI3, and VRIN. The cumulative photosynthetically active radiation was the real-time total photosynthetically active radiation value at the sensor monitoring location, which was calculated using the numerical integration method (Equation 1):


(1) $$R=\sum_{i=0}^{n}\frac{1}{2}\left(r_{t_{i1}}+r_{t_{i2}}\right)\left(t_{i2}-t_{i1}\right)$$

where R is the cumulative photosynthetically active radiation at the location of the monitoring point (sW/m^3^), $r_{t_{i1}}$ is the total active photosynthetic radiation at a time point $t_{i1}$ (W/m^3^), and $r_{t_{i2}}$ is the total active photosynthetic radiation at $t_{i2}$ (W/m^3^).

The distribution of cumulative photosynthetic active radiation in the greenhouse from south to north is closely related to the distance to the center of the greenhouse and the angle of solar radiation. The cumulative photosynthetic active radiation of the greenhouse was calculated and then used to calculate the cumulative photosynthetic active radiation value of each ridge using Equation 2:


(2) $$R_{N}=\text{a}\cdot \frac{{\text{t}}_{d}}{t}R^{b\left(\frac{L_{N}-L_{C}}{L}\right)}$$

where $R_{N}$ is the cumulative photosynthetic active radiation value at the position of the Nth ridge in the east-west orientation (sW/m^3^), ${\text{t}}_{d}$ is the current time (s), t is the total time of the day (s), R is the cumulative photosynthetic active radiation value at the position of the monitoring point (sW/m^3^), $L_{N}$ is the distance from the center of the Nth ridge to the back wall (m), $L_{c}$ is the distance of the monitoring point from the back wall (m), L is the total length of the greenhouse in the north-south orientation (m), and a , b are constants, whose values vary in different greenhouses; therefore, these values must be obtained by actual calibration.

Furthermore, the decision threshold for photosynthetically active radiation (*RNmax*) was set for the intelligent variable irrigation system. When *RNtotal* = *RNmax*, the central control module initiates a program to calculate the crop water demand.

Second, the greenhouse environment sensor records real-time data, such as air temperature, humidity, and photosynthetically active radiation, and transmits them to the central control module. The user dynamically calculates the crop water requirement *INdemand* using *the Penman-Monteith (modified) formula* (Equation 3) and inserts the results into the central control module:


(3) $$ET_{0}\;(P-M\;modified)=\frac{0.408\Delta \left(R_{n}-G\right)+\gamma \frac{1713\left(e_{a}-e_{d}\right)}{t+273}}{\Delta +1.64\gamma }$$

where $ET_{0}$ denotes the reference crop emersion (mm·day^-1^), Rn denotes net surface radiation (MJ·m^-2^·day^-1^), G denotes soil heat flux (MJ·m^-2^·day^-1^), $e_{a}$ denotes the saturated water vapor pressure (kPa), $e_{d}$ denotes the actual water vapor pressure (kPa), Δ denotes the change of the saturated water vapor pressure curve (kPa·°C^-1^), γ denotes the wet and dry table constants (kPa·°C^-1^), and T denotes the average indoor air temperature (°C).

Third, when the cumulative photosynthetic active radiation (*RNtotal*) reaches the photosynthetic active radiation decision threshold (*RNmax*) and after the crop water demand, *INdemand* is calculated using *the Penman-Monteith (modified) formula*, the central monitoring module instructs the wireless valve control module to turn on the irrigation. If *RNtotal*< *RNmax*, the central control module checks whether *RNtotal* is equal to *RNmax*.

Fourth, the irrigation monitoring module checks the flow rate of the water meter and the main pipe pressure during irrigation. When the irrigation water consumption *INcapacity* = *INdemand*, the central monitoring module issues a stop irrigation instruction to the wireless valve control module and executes *QNstop*. If *INcapacity*< *INdemand*, the central control module continues to evaluate whether *INcapacity* is equal to *INdemand*. When there were multiple ridges of irrigation simultaneously, the irrigation-monitoring module monitored the pressure of the main pipe. If the pressure was deficient to ensure the uniformity of irrigation, the controller actively suspended part of the ridge irrigation to ensure the uniformity of the front and back ends of the drip irrigation belt.

Finally, when *RNtotal* = *RNmax*, *QNopen* was executed, irrigation was activated, the *RNtotal* was reset to zero, and the accumulation was restarted to begin the next cycle. Various farmed ridges were maintained under different light and heat conditions based on the intelligent variable irrigation systems, and the irrigation cycle and irrigation water consumption appeared distinct.

The core module of the above intelligent variable irrigation system is the central controller, which has multiple signal interfaces and can be used for various types of sensors. The main ways for the central controller to connect the sensor include a wired connection and a wireless connection, and the wireless connection includes an analog signal interface and a digital signal interface. The analog signal interface is standard signal acquisition (4-20 mA current, 0-5 V voltage), which can be connected to a soil moisture sensor, soil temperature sensor, total radiation sensor, and photosynthetically active radiation sensor. Digital signal interfaces include RS485 and SDI-12 to connect flowmeters, water level sensors, multi-profile soil moisture sensors, and digital water meters. This experiment used a photosynthetic active radiation sensor, the soil moisture sensor, and the digital meter through the digital signal interface for RS485 connected to the control cabinet. In addition, a wireless greenhouse sensor can be connected to the central controller via a wireless module. Therefore, this test builds an intelligent variable irrigation system that has extensive adaptability.

### 2.2 Validation of the intelligent variable irrigation system in lettuce cultivation experiments

The study site was located in Xiaotangshan Town, Changping District, Beijing, China (E 116° 20’ 26”, N 40° 07’ 03”, 50 m above sea level), which has Warm temperate humid monsoon climate. The experiment used a 45-m-long and 7.15-m-wide solar greenhouse sitting north-facing south, with a planting area of 182 m^2^ in the east-west ridge orientation. In the solar greenhouse, “*American Large Fast-Growing*” *Lactuca sativa L*. (*loose-leaf lettuce*) was planted. On August 1, 2022, lettuce was planted in two rows on one ridge at a spacing of 20 × 20 cm, with a density of 6.12 plants/m^2^. Before planting, 400 kg of organic fertilizer and 40 kg of compound fertilizer were applied to each ridge of the solar greenhouse. Drip irrigation tubes with a diameter of 16.2 mm, wall thickness of 0.38 mm, rated flow rate of 1.35 L/h, and drip head spacing of 0.3 m, were placed along the roots of each row to water the lettuce. The plants were harvested on September 9 after a 40-day growth period. The growth period was divided into seedling stage (8.1-8.23) for 23 days, rosette stage (8.24-9.02) for 10 days, and mature stage (9.03-9.09) for 7 days.

**Figure 3:**
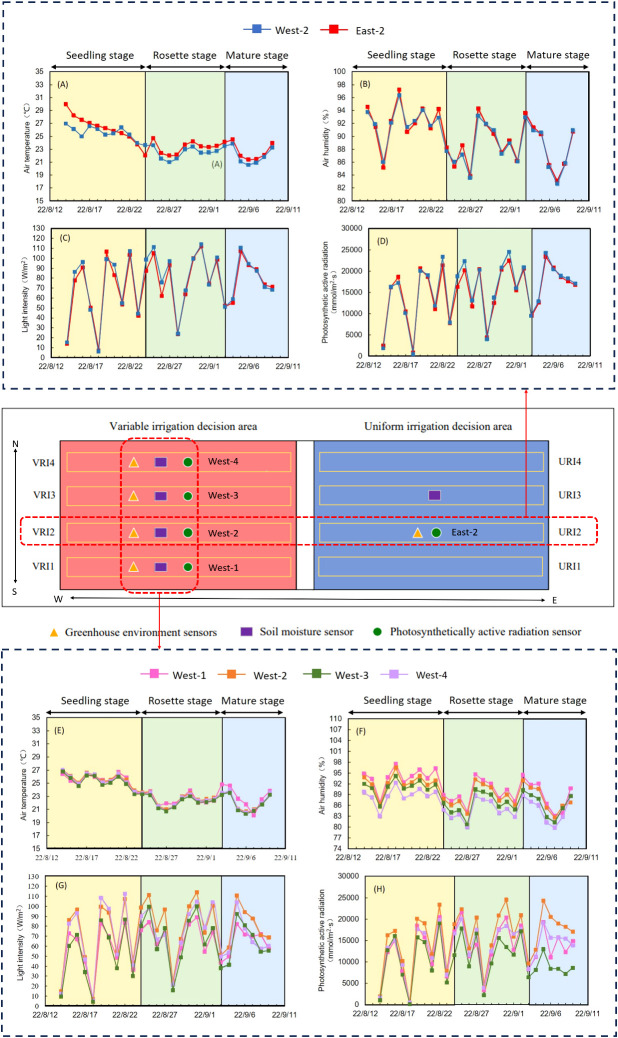
Environmental parameters at various locations within the solar greenhouse.

The solar greenhouse was separated into two water management regions in this study: the VRI region and the URI region. The VRI region differs from the URI region in that it has light heterogeneity under the east-west ridge planting in the solar greenhouse, and it was constructed to regulate the VRI region using a VRI method. As illustrated in **Figure 3**, four photosynthetically active radiation sensors were mounted on each of the four ridges to manage irrigation water consumption in the VRI region.

### 2.3 Data acquisition and analysis

#### 2.3.1 Data collection and analysis of the intelligent variable watering system

(1) An online environmental monitoring module included a greenhouse environment and photosynthetically active radiation sensors. A greenhouse environment sensor (greenhouse doll II, National Agricultural Informatization Engineering Technology Center) automatically monitored the environmental parameters in the solar greenhouse, such as air temperature, air humidity, and light intensity, such that it was always located above the crop canopy at a height of 20 cm, with a data collection frequency of 1 h/times. The photosynthetically active radiation sensor (RS-GH-I20-AL, Shandong Renke Measurement and Control Technology Co., Ltd.) automatically monitored the photosynthetically active radiation in the solar greenhouse, keeping it at the same height as the canopy and collecting data for 5 min/times.

(2) Irrigation monitoring included a wireless collector, water meter, and manometer. Irrigation water consumption was always recorded using an automatic water meter and a wireless collector (SM-10 electronic remote water meter, Nanjing Watergate Electronics Co., Ltd.).

#### 2.3.2 Data collection and analysis for crop growth

(1) The plant variables measured include plant height, plant width, number of leaves, leaf length, and leaf width. Six lettuce plants with uniform growth were selected and labeled at each locus. The above data were measured and recorded every five days from the end of the slow seedling stage to the end of the growth period.

(2) Photosynthetic parameters: During the rosette stage of lettuce development, single plants with uniform and robust growth and mature functional leaves in the same direction of light exposure were selected for measurement of photosynthetic parameters, it including the net photosynthetic rate (A), transpiration rate (E), stomatal conductance (gs), and intercellular carbon dioxide (Ci) by clipping the middle part of the leaves with transparent leaf chambers.

(3) The total yields of the URI and VRI regions were calculated, and then the VRI region was subdivided, and the total yield was calculated per ridge.

#### 2.3.3 Water use efficiency calculations

(1) The water use efficiency (WUE) was calculated using Equation 4:


(4) WUE=Y/ETc×100 %

where WUE denotes water use efficiency (Kg·ha^-1^·mm^-1^), Y denotes yield (kg·ha^-1^), and ETc denotes crop evaporative transpiration under standard conditions (mm).

(2) Irrigation Water Use Efficiency (IWUE) was calculated using Equation 5:


(5) IWUE=Y/I×100%

where IWUE denotes irrigation water utilization efficiency (kg·m^-3^), Y denotes yield (kg·ha^-1^), and I denotes irrigation water use (m^3^·ha^-1^).

#### 2.3.4 Statistical analysis

All statistical analyses were carried out using Excel 2016 and IBM SPSS Statistics 26 software. A one-way analysis of variance (ANOVA) was used to identify significant differences between treatment groups. P<0.05 denotes statistical significance. Finally, data visualizations were created in Excel 2016 and Origin 2022.

## 3 Results and analysis

### 3.1 Spatial and temporal patterns of environmental parameter changes in the solar greenhouse with the east-west ridge orientation

The fluctuation in environmental characteristics among different locations under the east-west ridge orientation is depicted in **Figure 3**. **Figures 3A–D** depict the environmental characteristics on the same ridge in an east-west ridge orientation. In contrast, **Figures 3E–H** depict the same axis in a north-south ridge orientation. The variations in air temperature, air humidity, light intensity, and photosynthetic active radiation in the solar greenhouse through the growth period ranged from 20.12–29.95°C, 79.78–97.5%, 3.91–114.12 W/m^2^, and 156–24,522 mmol/m^2^/s, respectively. The maximum light intensity and photosynthetic active radiation values occurred on August 31, 2022, with values of 2052.55 W/m^2^ and 424425 mmol/m^2^/s in West-2, respectively. The absolute differences in air temperature, air humidity, light intensity, and photosynthetic active radiation between East-2 and West-2 over the growth period were 0.71°C, 0.18%, 265.55 W/m^2^, and 12610 mmol/m^2^/s, respectively. However, none of the differences were significant in environmental parameters (P>0.05).

Except for air temperature, there were significant differences of changes in environmental parameters among the different ridges throughout the growth period (P<0.05). The average air humidity ranged were from, West-1 > West-2 > West-4 > West-3, with values of 90.88%>89.78%>88.03% >86.10% in descending order, respectively; the largest absolute difference was 4.78%. The cumulative light intensity and cumulative photosynthetically active radiation were West-2 > West-3 > West-1 > West-4, with values of 2052.55 W/m^2^>1905.10 W/m^2^>1641.24 W/m^2^ >1620.99 W/m^2^ >424425 mmol/m^2^/s >363607 mmol/m^2^/s >348661 mmol/m^2^/s >284,550 mmol/m^2^/s in descending order. The cumulative light intensity and cumulative photosynthetic active radiation showed that the center two ridges were higher than the two sides, with maximum absolute differences of 431.56 W/m^2^ and 139875 mmol/m^2^ s, respectively. West-2 exhibited the highest cumulative light intensity and cumulative active photosynthetic radiation during the growth period. The coefficients of variation of air temperature, air humidity, cumulative light intensity, and cumulative photosynthetically active radiation between different ridges were 7.99%, 4.10%, 40.58%, and 43.77%, respectively. Overall, the coefficient of variation value of cumulative photosynthetic active radiation was the highest when compared to air temperature, air humidity, and cumulative light intensity. The variability of cumulative photosynthetic active radiation was the highest among different ridges. Therefore, active photosynthetic radiation was used as an initiating factor for irrigation decisions in this study.

### 3.2 Water consumption analysis using variable irrigation decision-making methods

**Figure 4:**
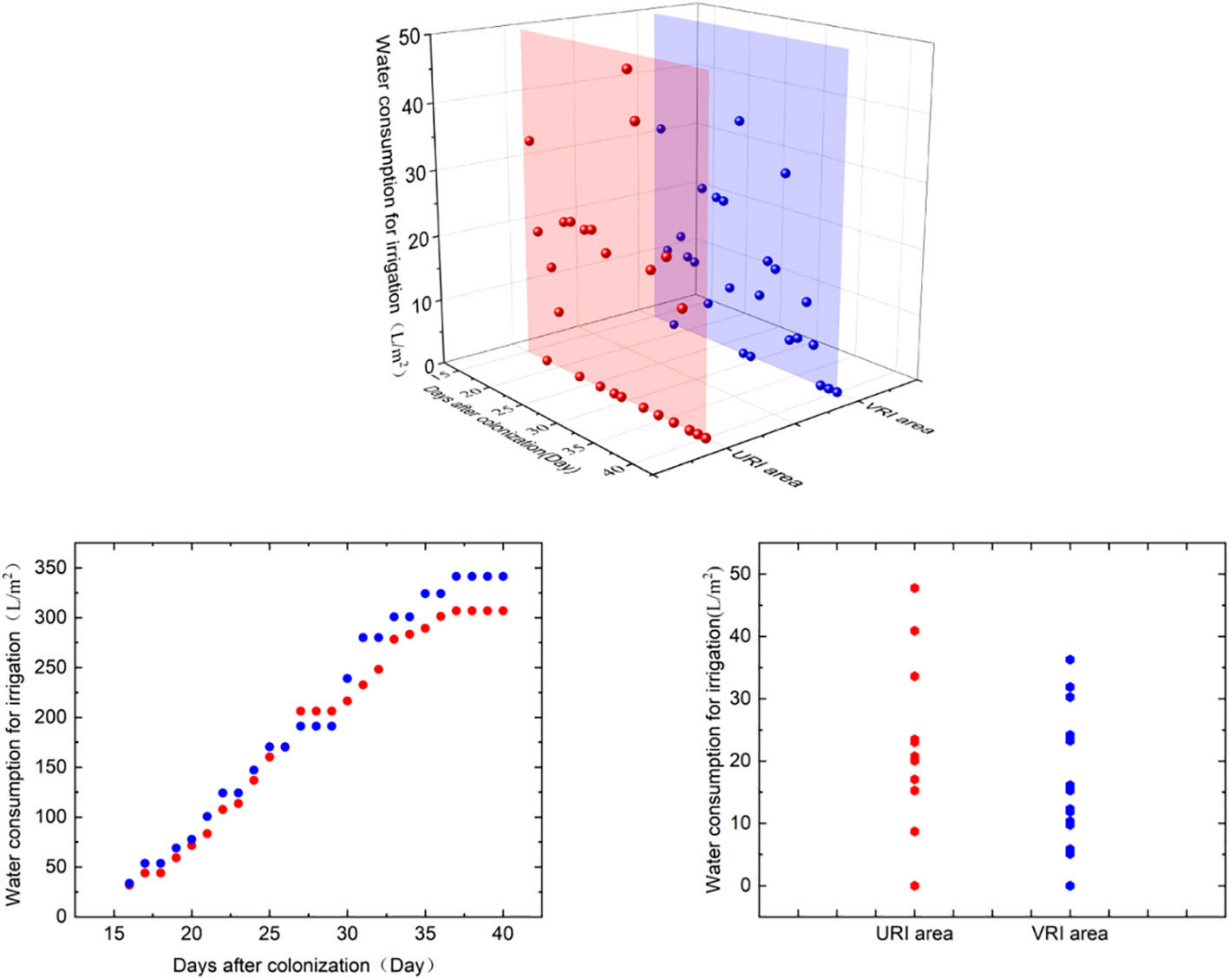
Irrigation water consumption in URI and VRI regions.

As shown in **Figure 4**, the total irrigation water consumption was 341.33 L/m^2^ and 307.12 L/m^2^ in the URI and VRI regions, respectively. Irrigation water consumption of VRI region was 10.02% less than URI region that was reduced by 7.39%, 14.12%, and 3.74% at the seedling, rosette, and maturity stages, respectively. This shows that the VRI method can reduce irrigation water consumption, which is most evident in the rosette stage.

**Figure 5:**
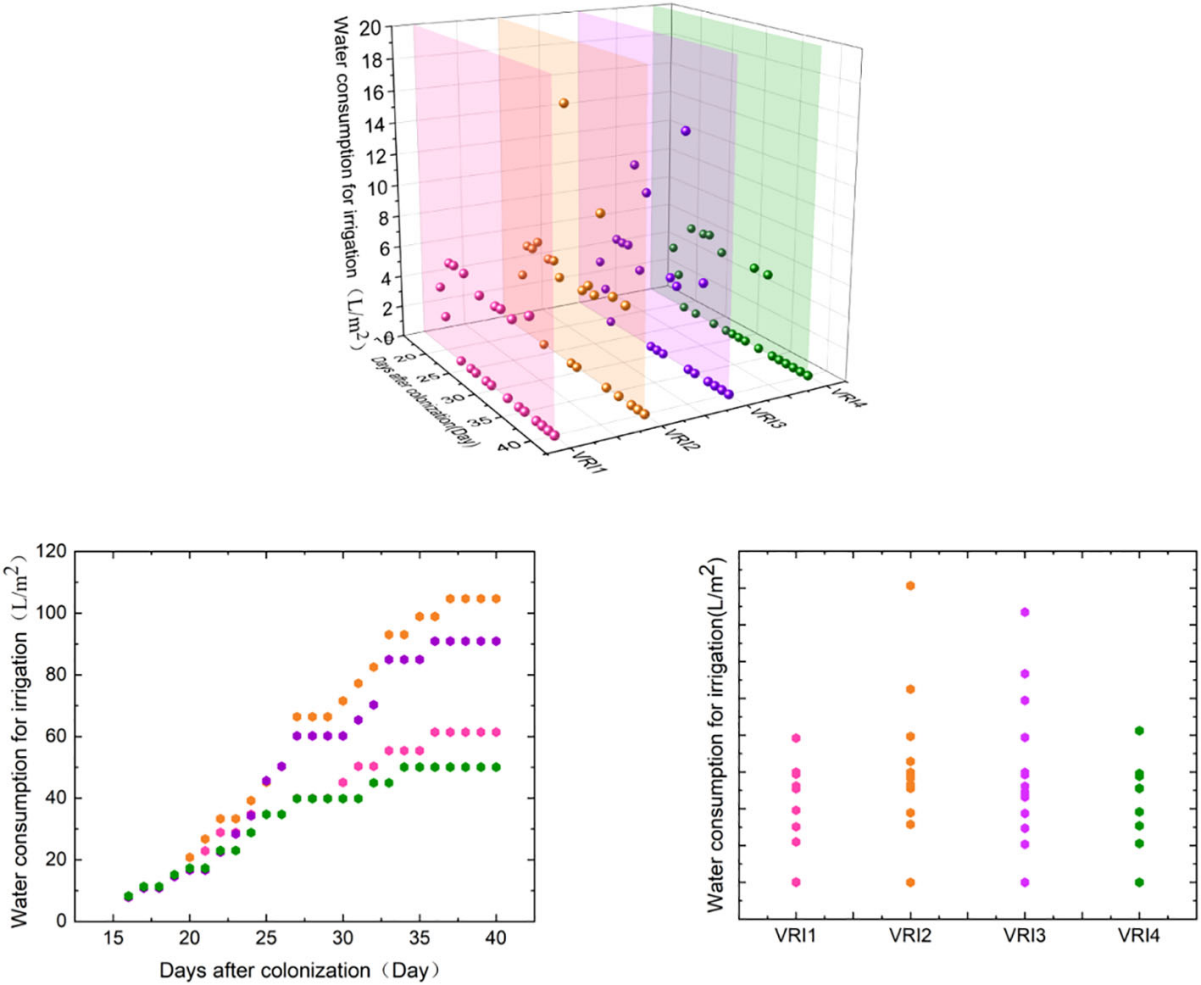
Water consumption for irrigation in the VRI region between different ridges.

**Figure 5** depicts the analysis of water consumption of different ridges in the VRI region. The irrigation water consumptions of VRI1, VRI2, VRI3, and VRI4 were 61.40 L/m^2^, 104.72 L/m^2^, 90.94 L/m^2^, and 50.06 L/m^2^, and the number of irrigation times was 12, 16, 14, and 10 times, respectively. The URI was compared with VRI2 and VRI3 irrigation water consumption increased by 22.72% and 6.57%, respectively. VRI2 and VRI3 of the number of irrigation cycles increased by two and did not increase, respectively. In contrast, the irrigation water consumption of VRI1 and VRI4 decreased by 35.07% and 41.33%, while the number of irrigation cycles decreased by two and four, respectively. The ranges of variation among different ridges within the seedling, rosette, and maturity stages were 23.03–33.31 L/m^2^, 21.93–59.73 L/m^2^, and 5.10–11.68 L/m^2^, respectively. The maximum absolute differences were 10.28 L/m^2^, 37.8 L/m^2^, and 6.58 L/m^2^, respectively, with the maximum difference in irrigation water consumption among different ridges observed during the rosette stage. The differences in irrigation water consumption among the different ridges proved the importance and necessity of a VRI method.

### 3.3 The impact of different VRI methods on crop growth and water utilization

#### 3.3.1 Impacts on crop growth

The response of the growth rate to the VRI method is discussed in **Table 2**. The absolute differences in the growth rates of plant height, plant width, number of leaves, and leaf area were 1.14%, 0.83%, 1.28%, and 1.12% between the URI and VRI regions, respectively. The absolute difference was slightly higher in the VRI region than in the URI; however, the difference was not statistically significant. The difference of the growth rates for plant height, plant width, leaf number, and leaf area was 35.34%, 30.98%, 48.64%, and 60.52% in VRI area of different points, respectively. The maximum absolute differences were 69.7%, 13.4%, 83.34%, and 308.02%. VRI2 and VRI3 benefited from adequate light and irrigation water, and their growth rates were higher than those of VRI1 and VRI4, resulting in inter-row growth rates among the different ridges.

**Table 2:** Variable irrigation-decision method affects growth rate and photosynthetic factors.

	Treatments		Between ridges			
Growth rate	URI	VRI	VRI1	VRI2	VRI3	VRI4
Plant height (%)	181.94	193.75	133.55	181.94	164.00	112.24
Plant width (%)	132.08	136.62	128.85	132.08	142.25	131.83
Number of blades (%)	250.00	266.67	216.67	266.67	240.00	183.33
leaf area (%)	512.45	557.55	458.39	512.45	659.60	351.58

The responses of photosynthetic parameters to the VRI methods are shown in **Table 3**. Throughout the growth period, photosynthetic metrics, including (A), (E), (gs), and (Ci), were measured on sunny and cloudy days. As indicated in **Table 1**, A and Ci increased by 23.2% and 8%, E and gs decreased by 4.44% and 11.28% in the VRI region under sunny conditions compared with those in the URI region, respectively. Under cloudy conditions, A, E, gs, and Ci all decreased by 8.9%, 5.9%, 7.46%, and 7.8% in the VRI region compared to those in the URI region, which suggests that VRI methods improve photosynthesis on sunny days and decrease photosynthesis on cloudy days. Further exploration of the photosynthetic parameters under sunny and cloudy states revealed that the maximum absolute differences for A, E, gs, and Ci were 1.05 μmol m^-2^ s^-1^, 0.85 mmol m^-2^ s^-1^, 315.16 mol m^-2^ s^-1^ and 1.6 μmol mol^-1^; 1.67 μmol m^-2^ s^-1^, 0.93 mmol m^-2^ s^-1^, 78.46 mol m^-2^ s^-1^, and 50.15 μmol mol^-1^ in VRI area of different points. The photosynthetic parameters of different ridges are thus quite different. VRI2 and VRI3 received sufficient light and irrigation water consumption; therefore, their photosynthetic activity was higher than that of VRI1 and VRI4, which led to larger disparities in photosynthesis and, thus, growth among the different ridges.

**Table 3:** Variable irrigation-decision method affect and photosynthetic factors.

	Photosynthetic parameters	URI	VRI	VRI1	VRI2	VRI3	VRI4
sunny	A(μmol·m^-2^·s^-1^)	3.06	3.77	3.21	4.26	3.98	3.62
	E(mmol·m^-2^·s^-1^)	2.25	2.15	1.83	2.68	2.09	1.99
	gs(mol·m^-2^·s^-1^)	731.24	648.79	474.08	759.24	789.24	572.62
	Ci(μmol·mol^-1^)	329.63	355.97	333	327.54	323.37	334.6
cloudy	A(μmol·m^-2^·s^-1^)	1.91	1.74	1.83	2.35	2.1	0.68
	E(mmol·m^-2^·s^-1^)	2.03	1.91	1.79	1.82	1.98	1.05
	gs(mol·m^-2^·s^-1^)	228.45	211.41	283.89	238.41	205.43	217.9
	Ci(μmol·mol^-1^)	397.82	366.87	385.66	377.12	369.17	335.51

#### 3.3.2 Impacts on yield, WUE, and IWUE

**Table 4** displays crop yield, WUE, and IWUE under the VRI method. Yield, WUE, and IWUE were higher in the VRI region than that in the URI region, with a significant difference (P<0.05). Specifically, yield, WUE, and IWUE grew by 9.12%, 12.00%, and 21.32% in the VRI region compared to those of the URI region, respectively. The yield and WUE of VRI1, VRI2, and VRI3 were greater than that in URI2, while the yield and WUE of VRI4 were 1.50% and 2.64% lower than that in URI2, respectively. The IWUEs of VRI1, VRI3, and VRI4 were higher than those of URI2; of these, the IWUE of VRI2 was 3.50% lower than that of URI2. The experimental results demonstrated that the VRI method improved crop yield, water use efficiency, and irrigation water use efficiency while reducing irrigation water consumption through controlled irrigation in ridges.

**Table 4:** Responses of yield, WUE, and IWUE to various irrigation-decision methods.

	Treatments		Between ridges			
	URI	VRI	VRI1	VRI2	VRI3	VRI4
Yield (kg·ha^-1^)	5365.04b	5854.07a	5797.44bc	6360.17a	5974.36ab	5284.31c
WUE(kg·ha^-1^·mm^-1^)	1245b	1391.47a	1617.06a	1423.69b	1352.72bc	1212.16c
IWUE(kg·m^-3^)	6.29b	7.63a	9.44b	6.07c	6.57c	10.56a

The same letter after the number indicates that there is no significant difference between different treatments, and different letters indicate that there is a significant difference between different treatments.

## 4 Discussion

Planting crops in the east-west ridge orientation can considerably enhance the efficiency of mechanized production. For example, Ningxia conducted an in-depth study on the mechanized cultivation mode of protected vegetables. The results showed that planting of the east-west ridge orientation promoted plant growth in a solar greenhouse. Further, researchers screened out suitable machinery for deep turning, rotary tillage, ridging, transplanting, stubble elimination, and inter-row operation. They observed that the mechanization rate increased from 29.0% to 67.8%, and the labor cost decreased by 8.1% (Yang et al., 2021a; Yang et al., 2021b; Yang et al., 2022). The light distribution in the solar greenhouse revealed that the light was concentrated in the middle of the greenhouse. In contrast, the wall obstructed the two sides, which limited the lighting. Further, the light distribution within the same ridge is not consistent in typical north-south ridge orientation planting, and there are many ridges, making it impossible to manage the ridges based on light distribution; however, east-west ridge orientation on the same ridge has fewer ridges, and it improved the consistency of the light and heat distribution. However, light and heat distributions differed between ridges, resulting in differences in crop growth based on ridge. The current method of average irrigation in solar greenhouses cannot meet the water demand of crops between different ridges, and there is excessive/insufficient water supply in some ridges, affecting crop growth and yield and lowering the efficiency of water use (Gundim et al., 2023). Therefore, based on the change in ridge orientation, ridge irrigation can be carried out according to the environmental differences in crop populations and water consumption.

**Figure 6:**
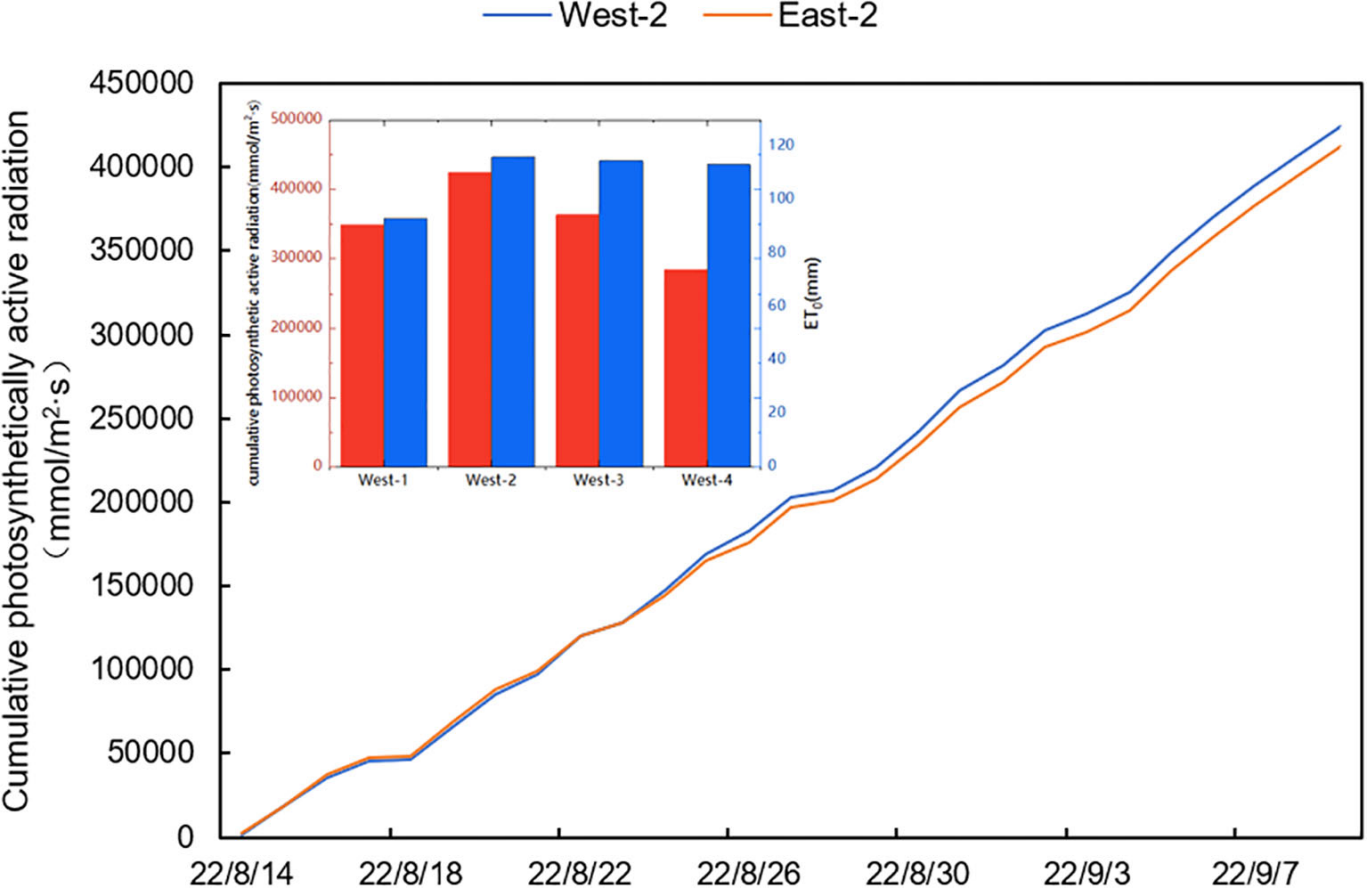
Total photosynthetically active radiation.

In this study, the differences of environmental parameters were significant (P<0.05) in the north-south orientation, except for in air temperature, where the coefficient of variation of photosynthetic active radiation was the highest; thus, it was selected as an initiating factor to regulate the time and amount of irrigation between different ridges. This pattern occurred because active photosynthetic radiation directly affects water consumption. In the production system, the water is absorbed by the crop root system in the soil and, under the influence of transpiration, passes through the stems to reach the leaves and then evaporates into the air, forming a dynamic water cycle system, the soil-plant-atmosphere continuum (SPAC) (Zhang et al., 2021). Photosynthetic active radiation is the main influencing factor of transpiration; when photosynthetic active radiation is strong, it promotes the movement of water in the soil and crop to the air, increasing crop water consumption. Conversely, weak photosynthetic active radiation slows the movement of water in the soil and crop to the air, thereby lowering crop water consumption, as shown in **Figure 6**. The cumulative photosynthetic active radiation in the solar greenhouse, which was West-2>East-2>West-3>West-1>West-4 over the growth period, indicating that the photosynthetic active radiation on the west side of the solar greenhouse was higher than that on the east side and confirming that the photosynthetic active radiation of the middle two rows was higher than that of the other two rows.

The validation experimental results demonstrated that irrigation water consumption decreased by 10.02%, yield increased by 9.12%, and WUE and IWUE increased by 12% and 21.32%, respectively, under the VRI method. A related study discovered that variable irrigation reduced total irrigation water consumption by 25%, increased yield by 2.8% and 0.8% for soybeans and corn, respectively, and increased water use efficiency by 31.2% and 27.1%, respectively. Therefore, the results of this study are consistent with those of previous studies. To conserve water, this study implemented an on-demand water supply in the sub-row, utilizing active photosynthetic radiation as the initiating factor. The main advantage of the VRI method for reducing irrigation water use is that it can accommodate the different resource allocation needs of multiple ridges on demand based on the received photosynthetically active radiation. The URI method, meanwhile, is based only on one ridge; thus, it ignores the difference in illumination between the different ridges.

**Figure 7:**
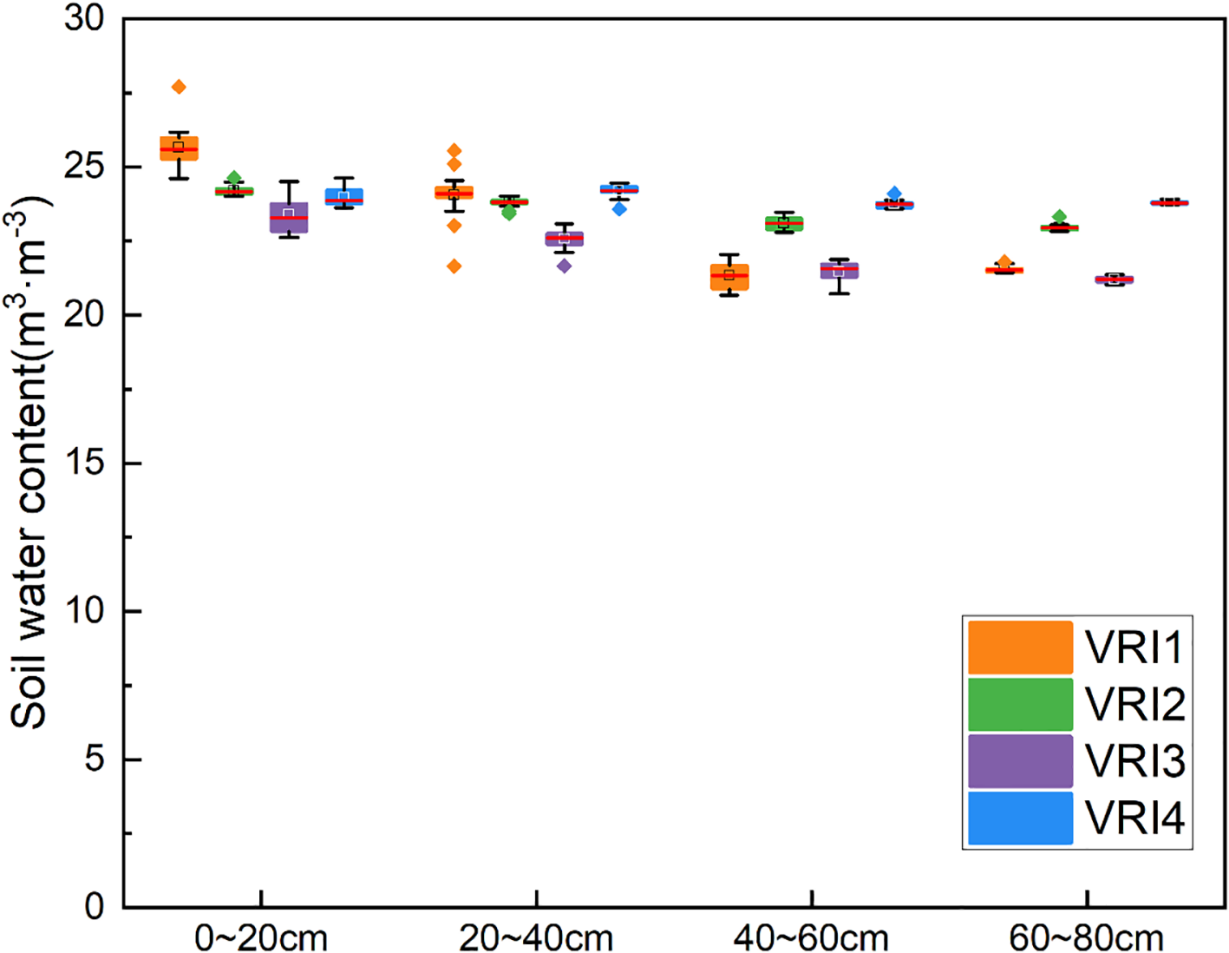
Soil moisture content varies between ridges in the T region.

A few studies have reported that uncontrolled or deficient water affects yield (Liu et al., 2022). In this study, change in irrigation water consumption led to an increase of 8.06%, 18.55%, and 11.36% in yields of VRI1, VRI2, and VRI3, respectively, compared with those of URI, except for the yield of VRI4, which was 1.50% lower than that of URI. The VRI method provides appropriate water consumption to increase yield. VRI1 had a higher yield with less irrigation water consumption, whereas the URI method may cause excessive water in certain areas, thereby reducing yield. WUE was influenced mostly by yield and improved in all treatments except for VRI4. Although irrigation water consumption was the most important factor determining IWUE, there was a negative correlation between IWUE and irrigation water consumption. Except for VRI2, IWUE increased in all treatments. According to a related study, grape growth and water status could be better controlled when there is a clear relationship between soil water content and ETa (Wilson et al., 2020). Therefore, in this study, the soil water content of the different ridges was analyzed. Pei Yun et al. reported that when the soil water content was 28.0–32.9 cm^-3^, the growth and photosynthesis of loose-leaf lettuce were optimal (Pei et al., 2015). Additionally, as shown in **Figure 7**, water homogeneity and on-demand supply among different ridges were achieved using the VRI method under inconsistent light conditions. Therefore, it can be concluded that the VRI method regulated by the intelligent irrigation system under east-west ridge-oriented planting is feasible for sustainable crop production, even under inconsistent conditions.

## 5 Conclusion

In this study, an IoT control system was used to develop a VRI method, and the effects of the VRI method were compared with those of URI methods. The results showed that the performance of the VRI method was better than that of the URI method in all respects. Compared to the URI method, the VRI method reduced irrigation water consumption by 10.02%, increased yield by 9.12%, increased WUE by 12%, and increased IWUE by 21.32. Additionally, using the VRI method, VRI1, and VRI4 obtained higher WUE and IWUE despite reduced irrigation water consumption, which verifies the advantage of variable irrigation. The results of this study may help produce solar greenhouses and obtain the most from limited water resources to achieve higher yields, WUE, and IWUE. Further investigations should be conducted on different light-sensitive crops, such as tomatoes and cucumbers, to verify the applicability of this method in other crop systems.

## Data availability statement

The original contributions presented in the study are included in the article. Further inquiries can be directed to the corresponding authors.

## Author contributions

LL: Writing – original draft, Writing – review & editing. FH: Writing – original draft, Writing – review & editing. JL: Methodology, Writing – review & editing. SA: Project administration, Writing – review & editing. KS: Formal analysis, Writing – review & editing. SZ: Methodology, Writing – review & editing. LZ: Conceptualization, Writing – review & editing.
